# Gastric Necrosis and Perforation Following Massive Gastric Dilatation in an Adolescent Girl: A Rare Cause of Acute Abdomen

**DOI:** 10.3389/fsurg.2019.00003

**Published:** 2019-01-30

**Authors:** Zlatan Zvizdic, Asmir Jonuzi, Aleksandra Djuran, Semir Vranic

**Affiliations:** ^1^Clinic of Pediatric Surgery, University Clinical Center Sarajevo, Sarajevo, Bosnia and Herzegovina; ^2^Department of Pathology, University Clinical Center Sarajevo, Sarajevo, Bosnia and Herzegovina; ^3^College of Medicine, Qatar University, Doha, Qatar

**Keywords:** gastric dilatation, necrosis, gastric perforation, pathological aerophagia, acute abdomen

## Abstract

Gastric necrosis with perforation is a rare and potentially life-threatening condition in childhood beyond the neonatal period. We report a case of gastric necrosis and perforation of a portion of the great curvature due to a massive gastric dilatation caused by pathological aerophagia in a 13-years-old, mentally impaired adolescent girl. Despite the successful surgical treatment, the patient's condition rapidly deteriorated post-operatively and she died due to the multisystem organ failure and multiple infections. In addition, we surveyed the literature on this rare condition and assessed the preventive actions to reduce this life-treating condition.

## Introduction

The stomach is a well-vascularized organ that receives a blood supply from the left gastric artery (a branch of celiac axis), the right gastric artery (a branch of the common hepatic artery), the right gastroepiploic artery (a branch of the gastroduodenal artery), the left gastroepiploic artery (a branch of the splenic artery), and the short gastric arteries, which also arise from the splenic artery. Therefore, gastric infarction is uncommon compared with other parts of the gastrointestinal tract. The reported causes of gastric infarction are diverse and include gastric volvulus (1), intrathoracic herniation (2), ingestion of caustic substances (3), complications after gastric surgery (4), arterial occlusion by embolus or thrombus (5), acute necrotizing gastritis (6), and massive gastric dilatation related to excessive food intake due to psychogenic polyphagia, pathologic aerophagia (PA), bulimia nervosa, and Prader-Willi syndrome (7–10).

Aerophagia, as one of the risk factors of gastric ischemia, represents a rare functional gastrointestinal disorder characterized by excessive and inappropriate swallowing of air resulting in progressive abdominal distension. The prevalence of aerophagia in children is estimated to be 7.5% (range 0.1–8.8%) (11–14). Although the disease is predominantly seen in healthy children exposed to stressful situations, 25% of the cases affects the children with severe psychiatric and/or neurological disorders (8). Aerophagia is usually short-term, but can become chronic in rare cases. If so, it can be associated with various gastrointestinal symptoms, such as abdominal pain, flatulence and belching when it is described as a pathologic aerophagia (PA) (15). In extreme cases, PA may progress to massive gastric and intestinal distension with the consequent development of ileus, volvulus, necrosis or perforation (16, 17). PA may induce the atony and hypotony of the alimentary tract, which in conjunction with chronic distension of the mucosa and smooth muscles of the gut, may lead to gastric or intestinal ischemia and consequent necrosis and perforation (17).

We report here a case of gastric necrosis and perforation due to massive gastric dilatation caused by PA in a 13-years-old mentally impaired girl. We also surveyed the literature on this rare condition and discussed the preventive actions that could hamper the fatal outcome.

## Case Report

**Figure 1:**
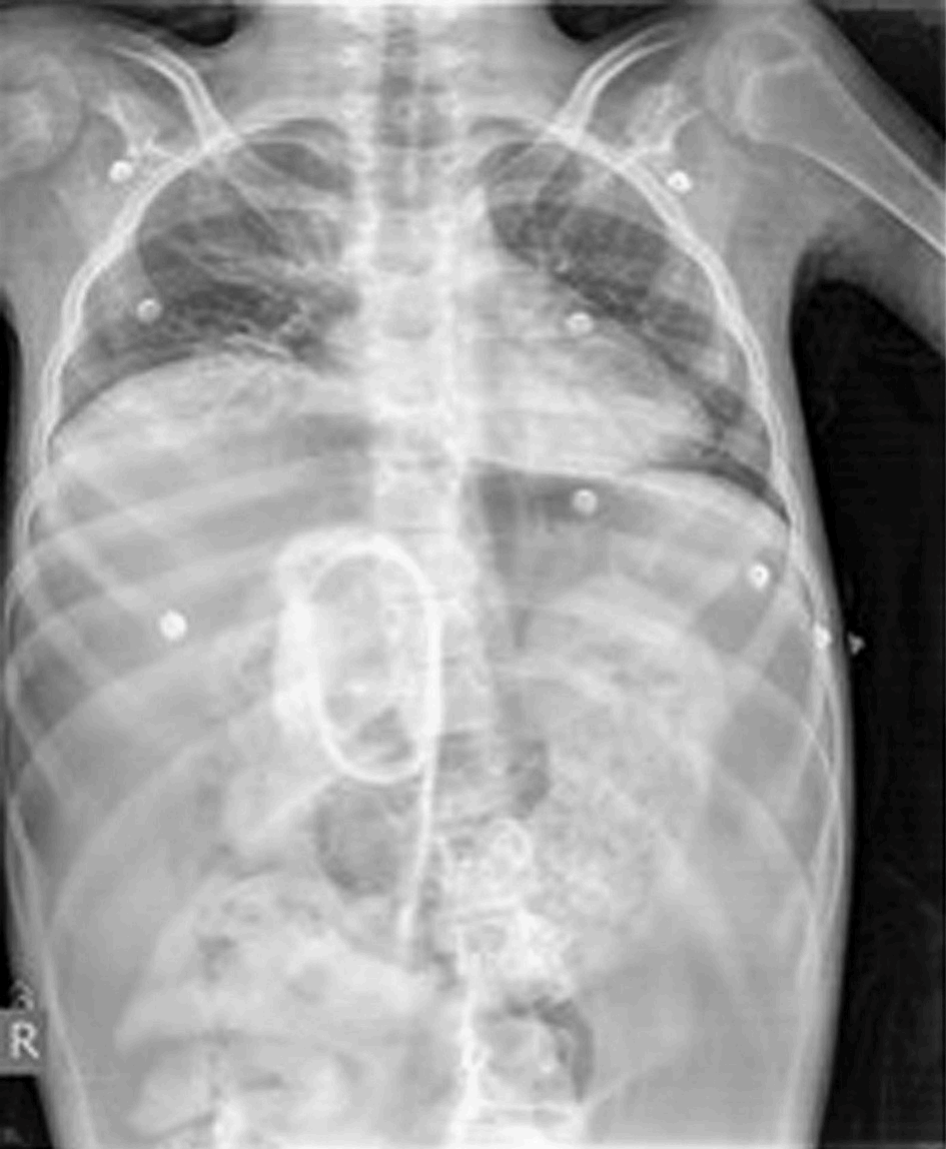
A plain abdominal radiograph showing a massive pneumoperitoneum within the abdominal cavity.

A 13-years-old mentally impaired girl (since birth due to perinatal asphyxia) presented to the emergency department (ED) with a severe abdominal pain and signs of acute abdomen, fever, and hypovolemic shock. She was unconscious, febrile, with a blood pressure of 80/40 mmHg, pulse rate of 160/min, and a respiratory rate of 34/min. Physical examination revealed a diffuse tenderness and a muscular rigidity. The abdomen was distended and bowel sounds were absent. The abdominal X-ray obtained in a supine position showed a massive free air within the peritoneal cavity and undigested remains of food along the alimentary tract imitating the contrasting liquid (Figure 1). At hospital admission, a medical audit accompanying the girl described a 4-days history of food rejection, frequent vomiting and progressive deterioration of her general condition. Her anamnesis was negative for traumatic events but was suggestive for PA due to a characteristic appearance of air swelling and abdominal distension that rapidly progressed during the day and caused the flatus during sleep.

Laboratory investigations revealed the following results: White blood cell count: 7.100/uL; mean platelet volume: 11.8 fL; platelet count: 220 × 10^3^/uL; hemoglobin: 11.1 g/dL; hematocrit: 35.2%; serum proteins: 5.7 g/dL; serum albumin: 2.9 g/dL; serum globulin: 2.8 g/dL; aspartate aminotransferase: 107 IU/L; alanine aminotransferase: 30 IU/l; creatinine: 0.9 mg/dl; serum sodium: 135 mEq/L; serum potassium: 5.4 mEq/L; serum chloride: 101 mEq/L; C-reactive protein: 367.4 mg/L; serum lactate dehydrogenase: 401 IU/L; serum creatine kinase: 4,086 IU/L; serum glucose: 107 mg/dL; arterial blood pH: 7.07 nmol/L; PaCO_2_: 4.93 kPa; pO_2_: 5.59 kPa; HCO_3_: 10.5 mmol/L; base excess: 18.8 mEq/L.

**Figure 2:**
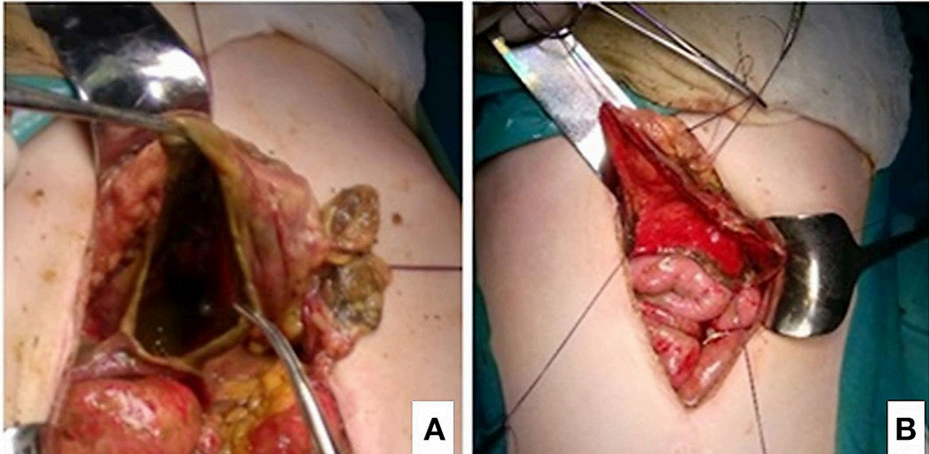
**(A,B)** Intraoperative findings of partial gastric necrosis and perforation (before and after debridement of necrotic tissue).

Following an aggressive resuscitation with intravenous hydration, decompression of the stomach, a correction of metabolic abnormalities, and administration of empiric antibiotic therapy (amikacin, metronidazole, and meropenem), an emergency laparotomy was performed. It revealed a massively distended and partially necrotic stomach. Huge amounts of free peritoneal fluid (about four liters) with food particles due to perforation of the necrotic gastric wall were removed. The size of the perforation measured 7 × 3 cm affecting the greater curvature on the posterior wall of the stomach (Figures 2A,B).

**Figure 3:**
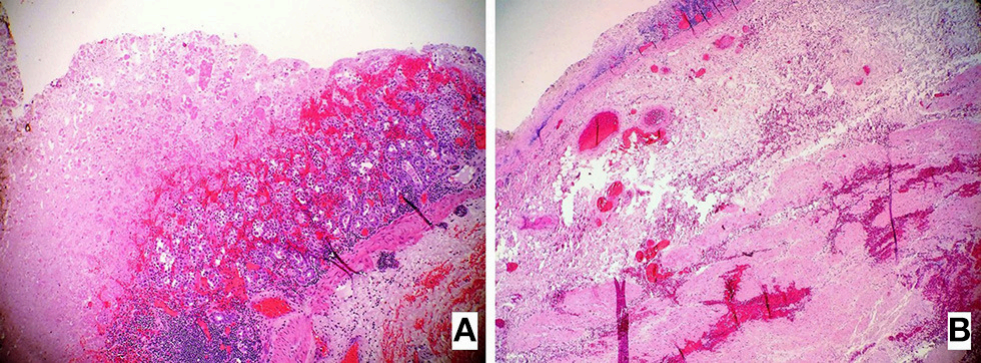
**(A,B)** Hematoxylin and Eosin-stained (H&E) sections of the resected stomach revealed the presence of the extensive necrosis affecting the entire gastric wall **(B)** while the left image **(A)** shows in part preserved the mucosal surface of the gastric wall (10×).

A free peritoneal fluid with food particles was washed out from the abdominal cavity. The gastric perforation was treated by debridement of necrotic tissue and a primary closure with additional using an omental patch. The debrided necrotic gastric tissue was submitted to the pathologist for the examination. The pathology assessment of the gastric wall showed a multiple areas of massive, transmural necrosis (Figures 3A,B).

Post-operatively, the patient was transferred to the pediatric intensive care unit (PICU) on mechanical ventilation and treated with inotropes, cefotaxime, metronidazole, gentamicin, and fluconazole. A fourth day of admission, abdominal cultures taken at the laparotomy as well as a blood culture showed *Enterococcus faecalis* and *Candida glabrata* infections.

In the following days, despite an extensive medical support, her clinical condition rapidly deteriorated and she eventually died on day 26 after admission due to overwhelming infections and progressive multisystem failure.

## Discussion

A gastric dilatation is a life-threating condition that may arise develop as a consequence of PA characterized by an excessive and inappropriate swallowing of air, especially in the mentally impaired persons (8). The resulting gastric dilatation may lead to a stomach necrosis and perforation. Aerophagia in association with mental illnesses constitutes 25% of all PA cases, particularly in patients with mental impairment, autism, and Rett syndrome (18). According to the Rome criteria, the diagnosis of PA is established in cases where abdominal distension and/or repetitive flatulence/belching, caused by air swallowing, presents for more than 12 weeks in a year (19). Gastric necrosis with perforation caused by a gastric dilatation is an extremely rare complication. According to some authors, gastric perforation occurs more frequently on the lesser curvature due to its reduced elasticity coefficient as well as its greater stretching compared with other parts of the stomach (20). In contrast, in the present case, gastric perforation affected the greater curvature. Patients with an acute gastric perforation require immediate resuscitation due to the third-space loss, and an immediate laparotomy is usually required. A surgical repair should be preceded by basic radiological procedures, such as an abdominal radiograph that would reveal a pneumoperitoneum, and an oral contrast application that would show an extravasation with a gastric wall irregularity or disruption (21). In children, gastric defects can usually be repaired with a simple closure, thus avoiding potential feeding difficulties seen after sleeve gastrectomy (22). We have applied such a procedure of simple suturing after the debridement of the surrounded thinned gastric wall. Unfortunately, after post-operative stabilization of the patient and a successful repair of the gastric defect proven by oral contrast, her clinical condition rapidly deteriorated and she died on day 26 after admission due to overwhelming infections and progressive multisystem failure. Gastric necrosis and perforation usually have a poor outcome with a recorded mortality rate of 50–80% particularly in cases of delayed diagnosis and treatment (23). In our case, the patient was admitted to the ED with signs of advanced peritonitis accompanied by metabolic disturbances, such as severe metabolic acidosis and hypotonic dehydration. A timely recognition of disturbed clinical condition, altered vital signs and laboratory findings suggestive of gastric perforation could have led to early hospital admission and prompt treatment that could save the patient.

The current preventive options of healthy children with PA consist of educating both the children and their parents and avoiding precipitating factors, such as drinking straws, carbonated drinks, and chewing gum. Psychotherapy may be particularly useful in children with associated psychological disorders. However, such approaches may not be suitable for children with severe mental impairment. Lee et al. (24) suggested the placement of a percutaneous endoscopic gastrostomy (PEG) to prevent massive and persistent bowel distention that could potentially result in surgical emergencies. Fukuzawa et al. (25) recently suggested a method of surgical bowel reconstruction (esophagogastric separation and abdominal esophagostomy via jejunal interposition) to solve the extreme forms of PA in the neurologically impaired children. None of these preventive measures (conservative or surgical) was undertaken in the presented case. We point out here that pediatric PA can be diagnostically challenging, especially in mentally impaired children due to the difficulties related to the clinical assessment (medical history and clinical examination).

In conclusion, this case shows the crucial importance of the preventive actions, early diagnosis and appropriate treatment of a gastric perforation caused by a PA. The preventive measures may be particularly challenging in mentally impaired children with PA as seen in our case. Nevertheless, the prevention of PA is worthy as it may hamper the potentially fatal consequences.

## Ethics Statement

The study has been performed in accordance with the ethical standards laid down in the 1964 Declaration of Helsinki. The informed consent was obtained from the patient's family. The case report was shared with the local ethical committee; it is however the policy of this committee not to review case reports. The patient's parent gave the consent to include the data in the present case study.

## Author Contributions

ZZ and SV conceived the study. ZZ and AJ did the clinical exam and performed the surgery. AD and SV did the histopathological diagnostics. All authors wrote and approved the final version of the manuscript.

### Conflict of Interest Statement

The authors declare that the research was conducted in the absence of any commercial or financial relationships that could be construed as a potential conflict of interest.
